# A comprehensive review of auditory verbal hallucinations: lifetime prevalence, correlates and mechanisms in healthy and clinical individuals

**DOI:** 10.3389/fnhum.2013.00367

**Published:** 2013-07-16

**Authors:** Saskia de Leede-Smith, Emma Barkus

**Affiliations:** Department of Psychology, University of WollongongWollongong, NSW, Australia

**Keywords:** auditory hallucinations, hallucinations, psychosis, schizophrenia, non-clinical, schizotypy, child, adolescent

## Abstract

Over the years, the prevalence of auditory verbal hallucinations (AVHs) have been documented across the lifespan in varied contexts, and with a range of potential long-term outcomes. Initially the emphasis focused on whether AVHs conferred risk for psychosis. However, recent research has identified significant differences in the presentation and outcomes of AVH in patients compared to those in non-clinical populations. For this reason, it has been suggested that auditory hallucinations are an entity by themselves and not necessarily indicative of transition along the psychosis continuum. This review will examine the presentation of auditory hallucinations across the life span, as well as in various clinical groups. The stages described include childhood, adolescence, adult non-clinical populations, hypnagogic/hypnopompic experiences, high schizotypal traits, schizophrenia, substance induced AVH, AVH in epilepsy, and AVH in the elderly. In children, need for care depends upon whether the child associates the voice with negative beliefs, appraisals and other symptoms of psychosis. This theme appears to carry right through to healthy voice hearers in adulthood, in which a negative impact of the voice usually only exists if the individual has negative experiences as a result of their voice(s). This includes features of the voices such as the negative content, frequency, and emotional valence as well as anxiety and depression, independently or caused by voices presence. It seems possible that the mechanisms which maintain AVH in non-clinical populations are different from those which are behind AVH presentations in psychotic illness. For example, the existence of maladaptive coping strategies in patient populations is one significant difference between clinical and non-clinical groups which is associated with a need for care. Whether or not these mechanisms start out the same and have differential trajectories is not yet evidenced. Future research needs to focus on the comparison of underlying factors and mechanisms that lead to the onset of AVH in both patient and non-clinical populations.

Auditory verbal hallucinations (AVHs) are a sensory experience that takes place in the absence of any external stimulation whilst in a fully conscious state (Beck and Rector, 2003). AVH occur with a sufficient similarity to the real percept that the individual attributes the event to be out of his/her own control (David, 2004). To date, the mechanism and pathophysiology of AVH, although widely speculated upon, are still largely unknown. The initiation and maintenance of AVH need to be distinguished and both explicated in order to begin to separate clinically relevant from protective factors for a differentiated trajectory of hallucinatory experiences. The current review aims to examine the phenomenology of AVH. We will consider the literature and data available across the lifespan as well as in different clinical and non-clinical groups. Extrapolating differences between clinical and non-clinical hallucinatory experiences provides an understanding of different developmental trajectories, characteristics of the experience and modes of interpretation for the voice hearer. As such, a review is timely which investigates the similarities and differences between the pathological voice hearing experience and AVH which are considered otherwise healthy modes of functioning. By integrating research in this very much evolving field, we can move forward toward a conceptualization of the intricate mechanism(s) responsible for the voice hearing experience.

The framework used in the current review is summarized in Figure 1. The biopsychosocial model provides a system where triggers, maintaining and moderating factors can be incorporated informatively. The domains interact with one another on a causal and mechanistic level, demonstrating the etiological complexity of AVH at any point along the lifespan and in both clinical and non-clinical groups. Factors can be conceptualized as background factors which are stable, may be biologically underpinned, and provide a backdrop against which other factors interact. These interacting factors can be mechanisms or triggers, the former contributing to maintenance and the latter initiating onset. However, the relationships between these variables are not discrete, the content of AVH can be informed by social and personal experiences. For example, the triggering environmental stressor can provide information for AVH content. This creates an intricate picture. However, given the complexity of the AVH experience it is not surprising that the factors which both initiate and maintain AVH are multifaceted and not mutually exclusive.

**Figure 1:**
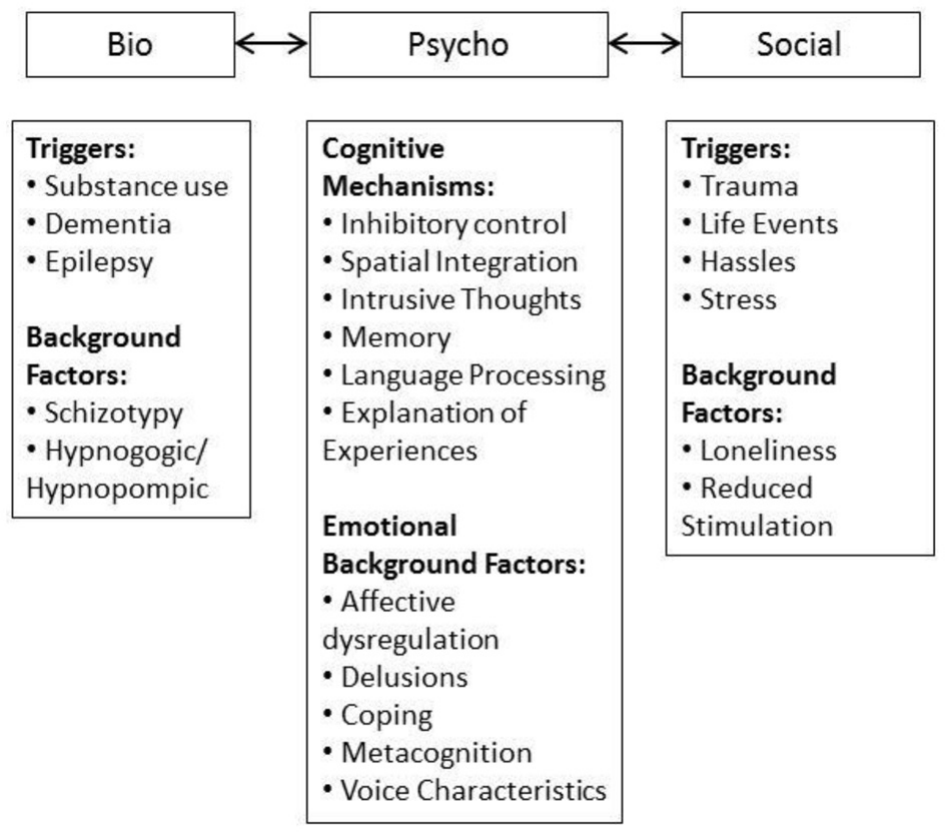
**Biopsychosocial framework used in the summary of the AVH literature**.

The principles of a systematic review were adopted in completing this literature review. The databases used included PsycInfo, Medline and Science Direct. Only peer reviewed journals were selected and analysed in the review process. However, it must be acknowledged that in all instances the most recent literature was focused upon, given that each of these sections would be deserving of a lengthy systematic review independently from one another. The search terms for each individual sub topic have been listed in brackets at the beginning of the corresponding section.

## Prevalence of AVH and related phenomena

### (Child, childhood, adolescent, adolescence, early adulthood, adult, auditory hallucinations, auditory perceptions, voice hearing, psychotic experiences, psychosis, clinical, non-clinical)

AVH are at their most prevalent in diagnosed psychotic disorders such as schizophrenia and schizoaffective disorder (Sartorius et al., 1986) but also occur in other disorders including bipolar disorder, substance intoxication and organic dementias. Recent research has focused on the existence of AVH in general population samples (Moritz and Larøi, 2008; Sommer et al., 2010; Daalman et al., 2011a,b; Temmingh et al., 2011; Larøi et al., 2012; Stanghellini et al., 2012). Epidemiological studies have estimated the prevalence of AVH to be between 5 and 28% in the general population (Tien, 1991; van Os et al., 2000; Johns et al., 2004; Scott et al., 2006). Johns et al. (2002) found 25% of individuals reporting hallucinatory experiences met the diagnostic criteria for a psychotic disorder; however that leaves 75% of people experiencing AVH who are considered otherwise healthy. Possible implications (which are by no means mutually exclusive) for the existence of non-clinical AVH are:

1. Healthy AVH may present as an isolated symptom and may not be related to any sort of predisposition for a psychotic disorder (Daalman et al., 2011a,b).
2. Alternatively, AVH may form part of a genetic predisposition toward psychotic illness. They can co-occur alongside other attenuated psychotic symptoms including paranoid ideation, odd/unusual behavior, delusions and inefficient cognitive processing (Krabbendam et al., 2005).
3. AVH are hypothesized to lie on a continuum of risk ranging from normal experiences to pathological psychotic (Johns and van Os, 2001) suggesting that clinically relevant AVH could be an extension of the processes occurring in otherwise healthy hallucinators.

### Childhood

AVH in children, like adults, are prevalent in both clinical and non-clinical populations (where clinical refers to those children diagnosed with schizophrenia or psychosis, unless stated otherwise). Studying the experience and trajectory of AVH in childhood provides insights into the development of these experiences from a biopsychosocial framework. Since the great majority of children reporting AVH never make the transition to psychotic disorder (Poulton et al., 2000), it seems factors mediate the likelihood of hallucinatory experiences becoming pathological. For example, it is often documented that biological factors such as pre- and peri-natal complications (Clarke et al., 2006; Zammit et al., 2009), along with delayed developmental milestones (Murray et al., 2004; Laurens et al., 2007) are associated with subsequent psychosis development. Examining the prevalence of child AVH in both clinical and non-clinical groups, a recent meta-analysis found rates to be between 5 and 16%, mostly occurring in late childhood or early adolescence (van Os et al., 2009). A prevalence of 8% for AVH has also been found in a cohort of 11-year old children (McGee et al., 2000), whilst Bartels-Velthuis et al. (2010) noted an almost identical prevalence of 9% for 7 and 8 year olds. Children also report musical hallucinations, although these are largely under-investigated and are often related to damage to the ear (Aziz, 2009). In children AVH are documented in conjunction with diagnoses of anxiety (Murase et al., 2000; Escher et al., 2002), migraines (Schreier, 1998) and depression (Ryan et al., 1987; Krabbendam et al., 2005; Scott et al., 2009) as well as conduct disorder (Kim-Cohen et al., 2003; Askenazy et al., 2007). In child clinical outpatients sampled by Askenazy et al. (2007) 100% met the required DSM-IV criteria for conduct disorder. Co-morbid conduct disorder is related to greater persistence and severity of AVH experiences (Kim-Cohen et al., 2003; Askenazy et al., 2007). Thus, the association between AVH and conduct disorders warrants further research. Interestingly, difficulties in metalizing (or theory of mind), particularly the identification of emotions in others, are common to conduct disorder (Sebastian et al., 2012), delusional ideation (Bartels-Velthuis et al., 2011a) and psychotic symptoms in general (Polanczyk et al., 2010). Potentially this provides a key point of overlap between these clustering of problems (see Bartels-Velthuis et al., 2011a).

It is clear that AVH are detectable in child populations. In most instances childhood AVH spontaneously cease: 76% of children who reported hearing voices aged 7 and 8 years stopped hearing voices by 12 and 13 years (Bartels-Velthuis et al., 2011b). Similarly, 75–90% of child psychotic-like experiences are transitory and regress over time (van Os et al., 2009). These studies imply that hearing voices may not be developmentally disadvantageous; particularly given the consistency with which they are reported to decrease with age (Escher et al., 2004; Askenazy et al., 2007; Bartels-Velthuis et al., 2011b). Further evidence for this can be drawn from the existence of imaginary companions which spontaneously cease, often when children begin school (Fernyhough et al., 2007). It has been reported that 46.2% of children between the ages of 5 and 12 years report the existence of at least one imaginary companion (Pearson et al., 2001). The experience of imaginary companions could be a young child's explanation for hearing voices (Pearson, 1998), although evidence does exist that children are able to distinguish between AVH and imaginary companions (Taylor et al., 1993). Having had or currently having an imaginary companion in childhood is not necessarily associated with negative mental health outcomes (Cohen, 1996; Taylor and Carlson, 1997; Hoff, 2005) and is not restricted in any way to those children with increased levels of creativity and imaginability (Pearson et al., 2001). Thus, the presence of an imaginary companion could be part of the normal development and sociability of the child, however further research in this area is warranted to understand their developmental relevance and long term outcomes.

### Adolescence and adulthood

Studies examining the prevalence of non-clinical auditory hallucinations in adolescents are limited compared to child and adult investigations. This is unusual given that the onset for prodromal symptoms for psychosis and other mental health disorders often emerge during mid- to late adolescence. Adolescence is the onset of a series of rapid changes in hormones and brain development. From a biopsychosocial perspective these changes are often cited as a possible explanation for the initiation and presentation of mental health symptoms which can evolve into schizophrenia. The brain's connections are at their greatest during adolescence (Ihara et al., 2009) before pruning and decreasing neuronal connectivity reduces them to adulthood levels (Hoffman and McGlashan, 1997). The stress associated with these changes has previously been cited as a trigger for psychopathology in certain individuals (Corcoran et al., 2003). The range of neurological, emotional and social changes which take place in adolescence may put predisposed individuals in a heightened state of vulnerability to psychopathology.

Estimated prevalence rates for adolescent AVH are similar to that of children, lying between 5 and 16% (van Os et al., 2009). Healthy adult voice hearers report beginning to hear voices at an approximate age of 12 years, which is significantly younger than their clinical counterparts (approximately 21 years; Tien, 1991; Daalman et al., 2011a). Some authors have proposed that aberrant synaptic pruning accounts for the onset of hallucinations (Hoffman and Hampson, 2011). However, given the consistency in the estimates for adolescent AVH and child AVH it is unlikely that this explains all cases. The resolution of childhood AVH coincides with early adolescence (Bartels-Velthuis et al., 2011b). There are a small subset of individuals who begin to re-experience an imaginary companion during adolescence (Barrett and Etheridge, 1992, 1994). However, these individuals have not been investigated to differentiate them from their counterparts or for the persistence of the imaginary friends during adolescence. The existing literature does not yet provide us with sufficient information to determine whether imaginary companions are comparable to AVH. This requires further investigation.

The prevalence of voice hearing in adult non-clinical populations is roughly the same as that in children, ranging from 10 to 15% (Tien, 1991; Sommer et al., 2010). The most common experiences reported by non-clinical adults take place on average every 3 days, for 2–3 min, are controllable for around 60% of the time and cause little to no distress or disruption to daily life (e.g., Daalman et al., 2011a). However, there do seem to be some healthy individuals who experience hearing voices to the same frequency and qualities as clinical patients with schizophrenia (Honig et al., 1998; Faccio et al., 2012). Given that the majority of childhood AVH resolve prior to adolescence, the rates in adulthood suggest that there are a significant group of individuals who develop hallucinations during adolescence and early adulthood which persist onward.

## Persistence of AVH

Persistence of AVH in childhood is reported to be indicative of a more severe underlying pathology (Bartels-Velthuis et al., 2011b). Under the biopsychosocial framework of AVH development, it is evident that there are certain mechanisms which contribute to the maintenance of hallucinatory experiences past the stage of initial development. A specific factor associated with the persistence of hallucinatory experiences in children is the formation of secondary delusions (Escher et al., 2002; Krabbendam et al., 2004; Freeman et al., 2010). The formation of delusions may be due to aberrant salience, or attributed importance, to AVH (Kapur, 2003). Delusional ideation is more likely to occur under situations of affective dysregulation; affective dysregulation in and of itself has been linked to the formation of psychotic symptoms in adult populations (Smith et al., 2006; Myin-Germeys and van Os, 2007; Bentall et al., 2009), warranting the need for investigation in children and adolescents. When states of anxiety, depression and stress interact with pre-existing hallucinatory phenomena affective disturbances can culminate to create delusional pathology (Krabbendam et al., 2005). Some authors have suggested that the combination of secondary delusions and emotional factors provides the mechanism for healthy AVH to become pathological experiences (Bartels-Velthuis et al., 2012). The need to find an explanation for the, often, unusual nature of AVH seems a logical necessity for human behavior. However, given that children are not necessarily bound by conventions of thought and social desirability (Osório et al., 2012) it is less clear why the ideas formed become unusual, fixed and associated with distress (delusional ideation). In 9–11 year olds, delusional-like ideas identified in children have been associated with more severe symptomatology (Laurens et al., 2012). It suggests that the distress associated with AVH is not necessarily socially bound and may be inextricably linked within the nature of the experiences.

In childhood the incidence of AVH has been reported to be a risk for a later transition to schizophrenia (Fenning et al., 1997; Poulton et al., 2000; Dhossche et al., 2002; although see Garralda, 1984). Besides psychosis, the presence of childhood AVH are concurrently associated with depression and anxiety (Murase et al., 2000; Escher et al., 2002; Krabbendam et al., 2005; Smith et al., 2006; Best and Mertin, 2007). They are also predictive for the later onset of depression, anxiety, paranoia, and bipolar disorder as well as psychosis (Bentall et al., 2012; Goghari et al., 2012; Goldstone et al., 2012; Smeets et al., 2012). Similarly, the presence of AVH during early or mid-adolescence has been associated with a substantially increased risk for a diagnosis of schizophrenia-spectrum disorders in their early twenties (Poulton et al., 2000; Welham et al., 2009).

Rates of discontinuation of AVH in adolescence have been reported to be between 3 and 40% each year (Rubio et al., 2012). Therefore, the significant minority for whom AVH persist during adolescence represent a distinct group (e.g., De Loore et al., 2011; Rubio et al., 2012). The persistence of AVH may be more likely to precipitate the need to generate explanations of the experiences as outlined above. The persistence-impairment model (van Os et al., 2009), suggests that the progression to increased impairment from psychotic-like experiences occurs at a point where the individual is exposed to sufficient environment stressors. The increasing independence required to navigate adolescence successfully would present many opportunities for increasing environmental stressors of a social (e.g., peer interactions, increased academic expectations) and biological (substance use) nature. Persistence of AVH in adolescence has been associated with increasing depression, general psychopathology, delusional ideation and need for care (Escher et al., 2004; Hanssen et al., 2005; De Loore et al., 2011; Dominguez et al., 2011; Mackie et al., 2011).

Often the investigation of AVH in adolescents coincides with the progression through to the prodrome for psychosis, bipolar disorder and other adult mental health difficulties. The development of the Clinical Staging Model (Wood et al., 2011) distinguishes residual symptoms or early signs in order for them to be detected more readily before progression to full psychopathology. The key factors implicated would be the persistence of the AVH, the presence of distress, other mental health symptoms and any type of help seeking behavior (Yung and McGorry, 1996). Since the majority of documented AVH in adolescence are considered to be non-pathological, there must be certain factors that impinge on the individual at some stage of their development to convert normal AVH to pathological problems requiring a need for care. Perhaps tracking these experiences over time, as well as the way in which they are interpreted, their qualities and the associated distress will help to highlight which child and adolescent AVH should be of concern to clinicians.

When examining the phenomenology of AVH in adolescents compared to healthy adult voice hearers, population based studies seem to illustrate a shared experience. Pearson et al. (2008) documented adolescent AVH which mirror those of adults, with these parallels being suggestive of a continuum of non-pathological hallucinatory experiences. The existence of such a continuum for healthy hallucinators progressing into adulthood could have functional benefits in relation to clinical staging assessments. Adolescence makes up a pivotal time of development where hallucinatory and other symptoms can progress to a prodromal stage requiring the first steps in a need for care. Clinical staging characterize disorders according to their seriousness, development and features. Such a conceptualization would be considerably useful if for example the individual's hallucinatory experiences were to progress past the initial stage to a more ingrained chronic impairment.

The consideration of prevalence and persistence of AVH leads to some consideration of the possible implications of AVH in the general population. That such symptoms exist within the general population now seems to be widely accepted within the literature. Additionally, the similarity in the levels in children, adolescence and adulthood implies that they do not necessarily confer developmental disadvantage. From the evidence considered so far, it appears that non-clinical AVH become pathological when they persist, lead to the development of other symptoms and cause distress and functional impairment. Accordingly, it could be argued that they do lie on a continuum of risk, ranging from normal healthy experiences, through to pathological psychotic (Johns and van Os, 2001). However, it cannot be determined whether the mechanisms which underpin non-clinical AVH are the same as those demonstrated in clinical AVH. Even though they may lie on a gradient of risk, this does not imply that the features present at the functioning end of such a spectrum mirror those at the extreme end of the spectrum. As has been presented, AVH are only associated with other attenuated psychotic symptoms when they require care. Therefore, non-clinical AVH in and of themselves do not seem to be indicative of the progression to mental health disorders. Additional clues to the developmental trajectories which differentiate clinical from healthy AVH can be derived from consideration of the phenomenology, cognitive mechanisms, and emotional regulation differences between the two populations.

## Comparison of clinical and non-clinical hallucinations in adult populations

### (Early adulthood, adult, auditory hallucinations, auditory perceptions, voice hearing, psychotic experiences, psychosis, clinical, non-clinical, phenomenology, schizophrenia)

In a comparison of the phenomenological features of child and adult voice hearers (Table 1), it is evident that components such as the localization, number of voices, and loudness of the voice hearing experience are largely consistent between clinical and non-clinical groups. Therefore, meaningful information can be derived by examining which features distinguish voice hearing in clinical groups from healthy voice hearers. Compared to AVH in schizophrenia (referred to as a “clinical” population in this section and including those with psychosis), non-clinical AVH have been found to occur much less frequently, and usually occur after specific conditions such as high stress or sleep deprivation (Larøi et al., 2012). The most commonly reported difference between healthy and clinical voice hearers is the emotional valence of the voice (Honig et al., 1998; Choong et al., 2007; Sommer et al., 2010), with a negative emotional appraisal of the voice having a predictive value of 88% for the presence of a psychotic disorder (Daalman et al., 2011a). Other phenomenological differences between the groups include a reduction in perceived control for psychotic AVH, as well as a higher frequency of AVH, and later age of onset (average of 21 years) when compared to healthy voice hearers (average of 12 years) (Daalman et al., 2011a). On the other hand, factors such as the loudness of the voice, attribution of source and perceived location all remain largely consistent between the groups, which is suggestive possibly of AVH differing primarily in terms of severity, rather than them being separate phenomena. Some authors have gone as far as to say that voice hearing may be adaptive for some healthy individuals (Faccio et al., 2012).

**Table 1:** **Phenomenological characteristics of AVH in clinical and non-clinical groups**.

**Adult AVH**		**Clinical (confirmed psychotic disorder) AVH**	**Non-clinical AVH**	**Able to distinguish between clinical and non-clinical groups?**
	Localization	Inside head (near ears) (Daalman et al., 2011a) Heard via the ears (78%) (Romme and Escher, 2000)	Inside head (further from body) (Daalman et al., 2011a) Heard via the ears (57%) (Romme and Escher, 2000)	No
	Explanation of origin	50% External (Nayani and David, 1996; Daalman et al., 2011a) Either inside or outside the head (hard to distinguish) (Nayani and David, 1996; Stephane et al., 2003; Copolov et al., 2004)	60% external, 40% internal (Daalman et al., 2011a) External source-mostly benevolent spirits (Sommer et al., 2010)	No
	Loudness	Little softer than own voice (Daalman et al., 2011a)	Little softer than own voice (Daalman et al., 2011a) 36% rated their voices as “normal” in loudness (Lawrence et al., 2010)	No
	Voices speaking in third person	50% (Daalman et al., 2011a) 39% (Romme and Escher, 2000)	25% (Daalman et al., 2011a) 27% (Romme and Escher, 2000)	Yes
	Controllability	20% of the time (Daalman et al., 2011a) 17% of the time (Romme and Escher, 2000)	60% of the time (Daalman et al., 2011a) 87% of the time (Romme and Escher, 2000)	Yes
	Number of different voices	11.44 (Daalman et al., 2011a)	7.62 (Daalman et al., 2011a) 51% heard only one voice (Lawrence et al., 2010)	Yes
	Frequency	One every hour (Honig et al., 1999; Daalman et al., 2011a)	One every 3 days (Honig et al., 1999; Daalman et al., 2011a) 25% heard their voices several times a day, 37% had not heard it lately (Lawrence et al., 2010)	Yes
	Duration	40 min (Daalman et al., 2011a) Continuous (Honig et al., 1999)	2–3 min (Daalman et al., 2011a)	Yes
	Types of voices experienced	Commenting voices (72%) (Romme and Escher, 2000)	Commenting voices (18%), voices speaking with each other (11%) (Sommer et al., 2010) Commenting voices (47%) (Romme and Escher, 2000)	Yes
	Mean age at first experiencing voices	21 years (Daalman et al., 2011a) 11% onset before 12 years (Honig et al., 1999)	14 years (Sommer et al., 2010) 12 years (Daalman et al., 2011a) 40% onset before 12 years (Honig et al., 1999)	Yes
	Disturbance to daily functioning	Moderate to severe distress, disruption (Daalman et al., 2011a) Significant disturbances to daily functioning (Honig et al., 1999) Disrupting daily life in 100% of voice hearers (Romme and Escher, 2000) Significant distress and disruption to the person (Evensen et al., 2011)	Disrupting daily life in 9% of voice hearers (Sommer et al., 2010) Almost no discomfort, disruption to daily life (Daalman et al., 2011a) Disrupting daily life in 20% of voice hearers (Romme and Escher, 2000)	Yes
	Emotional valence of voice	Majority of voices are unpleasant/annoying (Daalman et al., 2011a) 100% of voice hearers experience negative voices (Honig et al., 1999) (Romme and Escher, 2000)	4% of voice hearers experience negative content only (Sommer et al., 2010) Seldom unpleasant voices/content (Daalman et al., 2011a) 53% of voice hearers experience negative voices (Honig et al., 1999; Romme and Escher, 2000) Are evaluative of others but have mundane content (Leudar et al., 1997)	Yes
	Effect on individual	Frightening effect (78%); upsetting effect (89%) (Romme and Escher, 2000) Feelings of anxiety or depression (Freeman and Garety, 2003; Hoffman et al., 2008) 75% had moderate-severe anxiety ratings, 81% had moderate-severe depression ratings (Chadwick et al., 2000)	Frightening effect (none); upsetting effect (27%) (Romme and Escher, 2000) Over 50% fell within the normal range for anxiety and depression measures (Lawrence et al., 2010)	Yes
	Childhood trauma	33% Childhood sexual abuse (Honig et al., 1999) 53% childhood sexual abuse (Read and Argyle, 1999) 38% childhood sexual abuse (Offen et al., 2003) Experience of early trauma (Fowler et al., 2006) 75% experienced some sort of traumatic event (Escher et al., 2004)	Significantly more prevalent than healthy controls (Sommer et al., 2010)	No
	Family history axis I disorders	Increased risk of AVH in those who have biological relatives with the disorder (Erlenmeyer-Kimling et al., 1997; Aukes et al., 2008; Goldman et al., 2009)	Sig more prevalent than healthy controls (Sommer et al., 2010)	No
**Child AVH**	Localization	Similar to those in adults although not explicitly documented.	Inside their head (Best and Mertin, 2007) Attribution to an external source (Bartels-Velthuis et al., 2010)	No
	Number of voices	43.6% between 2 and 5, and 26% over 10 (Escher et al., 2004)	60% heard between 1 and 5 (Escher et al., 2004)	Partial overlap
	Frequency of voice hearing	20% hourly, 35% daily (Escher et al., 2004)	32% daily, 22% weekly (Escher et al., 2004)	Partial overlap
	Emotional valence	75% mainly negative (Escher et al., 2004)	33%+ heard unpleasant/threatening comments (Garralda, 1984) 47% mainly negative (Escher et al., 2004)	Yes
	Effect on the individual	10% associated anxiety/depressive symptoms (Escher et al., 2004)	Male voice: critical or threatening; female voice: helpful or supportive (Best and Mertin, 2007) 6% associated anxiety/depressive symptoms (Escher et al., 2004) 66% reported no/mild subjective burden (Bartels-Velthuis et al., 2010)	No
	Childhood trauma	Significantly more sexual and emotional abuse compared to healthy controls (Daalman et al., 2012)	100% from families with parents separated and domestic violence being a factor in many incidences (Best and Mertin, 2007) 1% reported sexual approach or abuse (Bartels-Velthuis et al., 2012) Sig more sexual and emotional abuse compared to healthy controls (Daalman et al., 2012)	No
	Family history axis I disorder	Heritability of schizophrenia, with certain abnormalities being trait markers for psychosis development (Weinberger and McClure, 2002; Cannon et al., 2003; Gilbert et al., 2003; Yucel et al., 2003)	50% family history of affective disorders (Garralda, 1984) Positive family histories for psychosis and depression (Burke et al., 1985)	No

This table provides a comparison of the characteristics of child and adult clinical and non-clinical voice hearers. It also outlines whether each phenomenological characteristic is able to distinguish between those with clinical and non-clinical AVH.

Apart from differences in those factors which may predispose individuals to experience AVH, there are a number of cognitive capacities which also distinguish clinical and non-clinical voice hearers, both of whom are distinguishable from healthy volunteers. These differences in cognitive capacities lend weight toward the view that there may only be a partial overlap in the healthy and clinical AVH experiences. Whilst the cognitive mechanisms may be detectable in the general healthy population, the degree to which they become “hard wired” responses used to process information from the environment may determine the development of other symptoms. The features which differentiate clinical and non-clinical groups, (namely those specific phenomenological characteristics and certain cognitive capacities) may be the key to understanding how AVH develop into a pathology requiring a need for care. For instance, the metacognitive component of low cognitive confidence was found to significantly predict auditory hallucinations (Morrison et al., 2007; Barkus et al., 2010). It is believed that difficulties in memory lead to fragmented retrieval, which in turn creates confusion and a lack of confidence for the individual (Morrison and Wells, 2003). AVH have been hypothesized to come about from a breakdown in the processes monitoring memory retrieval and the source of those memories (Seal et al., 2004). The link between hallucinations and memories (e.g., Bentall, 1990) and the possibility that hallucinations and intrusive thoughts share some commonality (Morrison, 2001, 2005) have been investigated in clinical and non-clinical AVH (e.g., Brébion et al., 1998, 2005; Moritz et al., 2001). Patients with schizophrenia who hallucinate have higher rates of intrusive thoughts than both non-clinical voice hearers and healthy volunteers (Morrison and Baker, 2000), whilst hallucination prone healthy volunteers reported more intrusive thoughts than low scoring counterparts (Jones and Fernyhough, 2006). The degree to which AVH impacts on the individual in terms of pathology is related in part to the individual's ability to understand, interpret and cope with intrusive thoughts (Lobban et al., 2002).

A cognitive factor that has been found to distinguish clinical from non-clinical AVH is inhibitory control. Inhibitory control and intentional cognitive inhibition specifically, is a reduction in the ability to inhibit the intrusive memories and thoughts discussed previously. Intentional cognitive inhibition has been specifically related to AVH above and beyond any other negative or positive psychotic symptoms (Waters et al., 2003). This poor inhibitory control has been replicated and extended in subsequent studies concerned with the prevalence and frequency of AVH in schizophrenia (Badcock et al., 2005; Soriano et al., 2009) and healthy individuals with high hallucinatory predisposition (Paulik et al., 2007). The relationship between AVH and intentional cognitive inhibition may be associated with executive resources in the prefrontal cortex (Badcock and Hugdahl, 2012). Whilst it seems that both clinical and healthy AVH groups have problems in inhibitory control along a gradient of severity (Waters et al., 2003; Paulik et al., 2007), Paulik et al. (2008) suggests the source of intrusions may be related to emotional dysregulation in non-clinical groups, whereas for clinical populations the source may relate more to impaired memory processes. This would account for the greater frequency of intrusions in clinical compared to non-clinical groups (Badcock et al., 2008; Daalman et al., 2011b).

The main feature which has been said to distinguish a person's normal thoughts from that of another voice (as in the voice hearing phenomenon) is the content of that thought. Most individuals with AVH hear sentences or words which they do not recognize as their own (Hoffman et al., 2008). The normal workings of inner speech for an individual usually change according to the pervasive mood of the person at the time, and also the situations which surround them (Langdon et al., 2009). Contrastingly, the content of AVH in diagnosed psychotic disorders usually reflects a more derogatory pattern of communication; characterized by a low linguistic complexity (i.e., the repetition of single words or phrases), usually through the form of accusation, command, or abuse (Nayani and David, 1996). The difference of their own thought patterns from those of AVH is one of the main reasons patients believe their thoughts stem from another source or location (Hoffman et al., 2008), with this process termed “alienation.” It is this non-self aspect which leads patients to believing such thoughts arise from external agents, such as; spirits, ghosts, deceased relatives or demons (Daalman et al., 2011a). However, the specific derogatory content of AVH in psychotic disorders has not been mirrored in studies involving the phenomenology of AVH in non-clinical populations. In such studies, the content of hallucinatory phenomena represents a more regular profile; either commenting on events taking place during the day, providing an evaluation of those around the individual or giving mundane utterances (Leudar et al., 1997; Romme and Escher, 2000; Sommer et al., 2010). In hardly any cases do non-clinical voice hearers report that the content of those experiences cause distress or dysfunction to their daily functioning (Sommer et al., 2010; Daalman et al., 2011a). When evaluating the voice hearing experience therapeutically, the content of voices is quite often ignored, with pharmacological interventions being the preferred method of treatment, rather than psychological. However, given the differences in the content of clinical vs. non-clinical hallucinatory phenomena, perhaps the content of voice hearing requires a greater degree of consideration in that which differentiates healthy experiences from pathological. It may be that the content of the voice is what drives the emotional appraisal of that voice, and as such represents an important phenomenological characteristic to be explicated upon in future research.

Compared to healthy non-voice hearers, higher levels of negative affect are common to AVH in schizophrenia (Delespaul et al., 2002) and otherwise healthy voice hearers (van't Wout et al., 2004; Allen et al., 2005) both during hallucinations and also when hallucinations are not present [for review see Freeman and Garety (2003)]. This is suggestive of emotional arousal possibly premeditating hallucination onset, or being a factor involved in the occurrence of these perceptual experiences (Slade and Bentall, 1988). Anxiety has the most predictive power for the predisposition to hallucinate in non-clinical groups (Paulik et al., 2006), over and above depression and stress ratings. Anxious non-clinical individuals have been shown to have a greater number of hallucinatory experiences (Allen et al., 2005), whilst in clinical voice hearers, there is a significant relationship between positive symptoms (hallucinations) and anxiety, rather than depression (Norman et al., 1998). Depression in clinical groups however, has been specifically associated with AVH of greater severity compared to their non-depressed counter parts (Smith et al., 2006). This points to a dynamic whereby higher depression ratings may be indicative of greater severity of the AVH to the individual, whilst higher anxiety is more strongly related to the level of distress those AVH illicit (Hartley et al., 2012).

Temporal contextual integration is one area dissimilar findings have been documented for healthy compared to clinical hallucinators. Performance on voice and location binding tasks is impaired in AVH with schizophrenia (Brébion et al., 2002; Chhabra et al., 2011) but not in hallucination prone healthy participants (Ruiz-Vargas et al., 1999; Badcock et al., 2008; Chhabra et al., 2011). This is indicative of differences in the ability to integrate spatial location cues for clinical vs. non-clinical groups, and suggests that a deficit in contextual integration does exist in psychosis specifically. Interestingly, the intact binding of memories for hallucination prone participants occurs specifically for the content and context (speaker identity) aspect of speech (Chhabra et al., 2012). This is suggestive of a dysfunction in the contextual integration of clinical hallucinators that has some sort of relation to the content or personal aspects of the memory itself. In related research, Bendall et al. (2011) were unable to demonstrate a deficit in the contextual binding of memories for individuals with first episode psychosis. This could be suggestive of memory binding dysfunctions only occurring when psychosis is completely developed, which may co-occur with the already demonstrated deterioration in memory function for these individuals (Frommann et al., 2011). As a result, deficits in contextual integration may be representative of a general vulnerability for psychosis, instead of specific to hallucination predisposition.

Another area of dissimilarity between clinical and non-clinical AVH groups concerns lateralization of language functions during verbal fluency tasks (Diederen et al., 2010). Decreased lateralization of language function has been well documented in schizophrenia literature [for review see Li et al. (2009)]. In healthy participants, verbal fluency tasks typically activate the prefrontal cortex in the left hemisphere, which has also been reported in healthy voice hearers (Diederen et al., 2010). This implies that the failure to establish left hemisphere dominance for language is not a specific mechanism that underlies AVH. However, it does not rule out the possibility that decreased language lateralization may be related to the pathological nature of AVH specifically, such as the frequency or negative emotional content which differentiates them from healthy hallucinatory experiences.

The type of functional coping strategy used to manage hallucinatory experiences is emerging as an important determinant of the risk of progression to pathological AVH. A tendency to suppress unwanted hallucinatory stimuli is associated with persistent and pathological hallucinations (Goldstone et al., 2012). Clinical voice hearers have been found to adopt passive strategies that do not allow control over their experiences (Larøi, 2012). In comparison, healthy voice hearers have been found to possess a feeling of control over their experiences through the use of problem solving, distraction and other active coping strategies (Larøi, 2012). This is just one of many vulnerability factors identified which may impact on the progression to pathological AVH and which require further research.

A comparison of the previously discussed phenomenological characteristics of AVH in adults and children, and across clinical and non-clinical groups has been provided in Table 1. When comparing information regarding the perceptual quality of the voice hearing experience in both child and adult populations, it can be seen that features such as the localization, number of voices, and loudness of the voice hearing experience are largely consistent between clinical and non-clinical voice hearers. Antecedent features which may be associated with the onset of the voice hearing experience also seem similar between clinical and non-clinical groups, regardless of age. This could point to common developmental trajectories for AVH in both groups, with similar environmental and biological factors associated with the onset of AVH. As a result it can be asserted that it is not the experience of voice hearing *per se*, or features predisposing AVH onset that are associated with psychological dysfunction. Why the developmental trajectories are “triggered” in some during childhood rather than adolescence has not been investigated. Given that younger age of onset of AVH seems to be associated with healthy voice hearing suggests this is worthy of investigation.

The most notable differences between healthy and clinical voice hearers seem to be the emotional valence of the voice and the distress voice hearing elicits. This seems to be particularly in regard to the controllability and the increased frequency of the experience for clinical voice hearers. These differences may stem from an interaction between:

1. Cognitive mechanisms: appraisal of the content; coping; thoughts/delusions related to the experience; and, inhibitory control;
2. Emotional regulation: appraisal of the emotional tone of the experience; metacognitive processes underpinning emotions and general metacognitive capacity. These dictate the emotional tone and loading of thoughts, specifically through experiential avoidance (Goldstone et al., 2012) or metacognitive beliefs in general (e.g., Varese et al., 2011).

One of the major cognitive mechanisms suggested as a component cause in the generation of AVH experiences is a lack of inhibitory control. Instinctively appealing, such a conceptualization satisfies the notion reported in many phenomenological studies of a reported lack of personal control over the generation and subsequent experience of voice hearing in both clinical and non-clinical groups. Impairments in intentional cognitive inhibition (the conscious active suppression of mental processes/thoughts) specifically have been put forward as factors linked to AVH experiences. This relationship is independent of any association to other positive, negative and disorganized symptoms of schizophrenia (Waters et al., 2003), demonstrating its specific association to AVH as a symptom unto itself. Intentional cognitive inhibition deficits follow a gradient of severity whereby non-clinical hallucinators demonstrate an impairment intermediate to clinical hallucinators (at the extreme) and healthy members of the general population (where little/no deficit exists) (Waters et al., 2003; Paulik et al., 2007). This relationship mirrors our observations of the phenomenology of clinical and non-clinical AVH experiences, lending to its significance in the generation of hallucinatory phenomena.

If deficits in intentional cognitive inhibition are implicated in the experience of AVH for all individuals, what component must interact with this dysfunction to create clinically significant AVH experiences in some people, but not in others? This difference is believed to lie in the way in which emotions are regulated, appraised and controlled for clinical vs. non-clinical groups. High levels of negative affect, primarily anxiety, depression and stress, have been documented both prior to and at AVH onset for clinical voice hearers [for review see Freeman and Garety (2003)]. Such emotional states are suggested to be involved in the development of the AVH rather than a consequence of it, as levels of negative affect have been found to fall (rather than rise) at the end of a hallucinatory episode, and increase immediately prior to an episode (Delespaul et al., 2002). So how is it that this dysregulation of emotion acts to create differences in the appraisal of AVH for clinical and non-clinical voice hearers? It has been put forward that high states of anxiety act to exacerbate deficits in intentional cognitive inhibition by increasing intensity above a critical threshold (Slade and Bentall, 1988) which act to create distressing intrusive thoughts (Paulik et al., 2006). Under this hypothesis, the individuals control over intrusive cognitive events is compromised even further by a heightened state of arousal which impairs that person's ability to function rationally and with clarity. It is also hypothesized that under this increased state of arousal, the individual's control regarding the feasibility of their metacognitive beliefs is compromised. Patients with AVH score higher on metacognitive beliefs in relation to uncontrollability and worry (Baker and Morrison, 1998). When these metacognitive beliefs occur in the context of AVH, they may act to exacerbate the negative emotional states which are already present as a result of AVH onset. The interplay between these beliefs and an already heightened mood state may dictate the appraisal of a negative emotional tone for the individual, and place emphasis on ways of thinking associated with paranoia, anxiety and distress. Although feasible, this line of reasoning requires further research before claims to its plausibility can be made.

An alternative line of research has suggested that the effectiveness of inhibitory control is dependent on limited, finite self-control resources (Baumeister et al., 2007; Goldin et al., 2008). In schizophrenia, suppression is used as an (ineffective) resource to deal with unwanted emotional expression (Henry et al., 2007, 2008). It has been put forward that the tendency to over use suppression to control unwanted emotions acts to deplete executive resources, the same of which control inhibition and are already reduced in schizophrenia (Gyurak et al., 2009). As a result, exacerbations in cognitive inhibition occur, which act to increase the severity and duration of AVH in clinical voice hearers (Waters et al., 2003). Thus, it could be argued that the reliance on emotional suppression to reduce unwanted emotions in clinical patients may result in a reduction of executive resources dedicated to inhibitory control. As cognitive inhibitory mechanisms have already been demonstrated as depleted in voice hearers, a subsequent reduction may act to increase the duration, frequency, and overall distress associated with AVH for clinical groups (Badcock et al., 2011). As this is only speculation at this point further research is warranted.

What seems to be pertinent to present research is the identification of features which allow these experiences to be dealt with in a beneficial manner. What strategies do non-clinical voice hearers adopt which allow them to regulate their experiences in an emotionally beneficial manner? It seems that they may possess coping strategies which allow them to deal with their experiences in the face of highly stressful or traumatic events. Research concerning the adaptive strategies of non-clinical voice hearers has suggested that an increased use of adaptive emotional regulation strategies (such as reappraisal) may allow the individual to adequately cope with the distressing nature of their experiences (Larøi, 2012). In contrast, clinical voice hearers have been found to use a greater number of maladaptive emotional regulation strategies (such as suppression) (van der Meer et al., 2009; Badcock et al., 2011). As a result, this leaves them in a position where they are unable to appropriately cope with their experiences, resulting in higher levels of distress and a negative emotional appraisal of the voice hearing experience. However, the precise mechanisms and processes which are involved in regulating the emotional appraisal associated with hallucinatory experiences has not yet been disseminated. As such, an understanding of these mechanisms is pertinent to the conceptualization of the differing developmental pathways leading to either: (a) clinically relevant AVH which cause distress and impairment, or; (b) healthy AVH experiences which allow the individual to function adaptively in society.

## Trauma and hallucinations

### (Child, childhood, adolescent, adolescence, early adulthood, adult, auditory hallucinations, auditory perceptions, psychotic experiences, voice hearing, psychosis, clinical, non-clinical, trauma, sexual abuse, abuse, intrusive thoughts, re-experiencing trauma, PTSD)

One of the most well researched triggers implicated in the pathway to AVH development is that of traumatic life experiences. Romme and Escher (1989) found that 70% of voice hearers sampled first began to hear their voices following a traumatic or significant emotional event. The traumas focused on in the existing literature can include severe trauma such as abuse, neglect, the loss of a parent or more commonly experienced traumas of childhood such as bullying and parental separation. Traumatic events which occur early on in development during childhood or adolescence are primarily cited. Perhaps traumatic events as environmental stressors during critical periods of development establishes voice hearing as a coping style or contributes to the development of cognitive mechanisms which may lead to voice hearing in adult life. Under this assumption, voice hearing becomes an adaptive process, yet the mechanism that makes this process pathological in some and functional in others is still unknown.

Trauma is implicated in the initiation and clinical relevance of child AVH (Escher et al., 2002; Morrison et al., 2003; Whitfield et al., 2005; Kelleher et al., 2008; Freeman and Fowler, 2009; McAloney et al., 2009; Elklit and Shevlin, 2011; Mackie et al., 2011). One of the largest areas of trauma associated with AVH is child sexual abuse (e.g., Janssen et al., 2004; Read et al., 2005). Although controversial, one group of researchers go so far as to claim causality (Read et al., 2005). McCarthy-Jones (2011) noted that 36% of clinical (which, as previously refers to those diagnosed with schizophrenia or psychosis) patients with AVH reported child sexual abuse, as well as 22% of healthy voice-hearers. Similarly, 56% of clinical patients who reported child sexual abuse also experienced AVH. The content and qualities of the voices heard has been linked to the identity of the abuser and verbalisations during the abuse (Read and Argyle, 1999; Read et al., 2003).

The emotional appraisal and nature of the abuse have been put forward as significant contributing factors. Associations of AVH in childhood have also highlighted the involvement of stressful life events (Bartels-Velthuis et al., 2012), daily stress (Myin-Germeys et al., 2005) and delusional ideation (Bartels-Velthuis et al., 2012) as other impacting factors. Traumatic experiences have been found to reflect the content of AVH indirectly in 58% of cases and directly in 13% of cases (Hardy et al., 2005). Therefore, it is possible that the relationship between AVH and child trauma operates through memory (see Bentall, 1990). However, it may be through a subjective interpretation of the trauma rather than directly through relived intrusive thoughts [(Morrison, 2001; Waters et al., 2006), and for a meta-analysis see Waters et al. (2012)]. Emotional distress associated with trauma may contribute to decreasing reality monitoring and increase the likelihood that memories and/or thoughts are attributed to an external source (Mertin and O'Brien, 2012).

Bullying is an emerging concern for contributing to the development of AVHs (e.g., Arseneault et al., 2010). Bullying in childhood leads to an approximately 2-fold increased risk for the presence of psychotic symptoms (Lataster et al., 2006; Schreier et al., 2009). This relationship existed for both subjective and independent reports (Schreier et al., 2009). Females reporting bullying at aged 8 years were associated with need for care and antipsychotic treatment in adulthood (Sourander et al., 2009). The emotional appraisal of the bullying and the development of secondary beliefs surrounding paranoia (which may be protective albeit maladaptive), have been implicated in reporting psychotic-like experiences following bullying (Campbell and Morrison, 2007). The capacity to experience AVHs in childhood and adolescence may co-occur with the expression of other symptomatology such as depression, anxiety or unusual personality traits such as schizotypy. These may mark a child or young person as “different” from their peers and therefore more likely to experience victimization (Turner et al., 2010). The factors which lead to the bullying, and indeed other traumatic events, need to be established since they may be indicative of psychopathology and potential confounds in the relationship between traumatic experiences and AVHs. This is particularly important when considering that experiencing one traumatic event as a child seems to increase the likelihood other traumatic events will occur (e.g., Finkelhor et al., 2007; Cuevas et al., 2010).

Like the aforementioned deleterious effects of trauma experienced during childhood, traumatic experience occurring and/or being re-experienced in adulthood can have similar psychological consequences. In one of the first epidemiological studies examining this relationship (Read et al., 2005), there were significantly more associations found between childhood maltreatment and voice hearing in adulthood, compared to any other symptom dimension (delusions, negative symptoms, thought disorder). This finding has been replicated in many subsequent investigations: the experience of AVH is significantly more likely to occur after psychological trauma in clinical (Read et al., 2003; Hardy et al., 2005; Romme and Escher, 2006, 2010; Reiff et al., 2012) and non-clinical (Honig et al., 1998; Shevlin et al., 2007; Kelleher et al., 2008; Sommer et al., 2010) groups. Therefore, the experience of trauma in and of itself does not constitute a good distinguishing factor between clinical and non-clinical groups. However, in an exploratory study, clinical and non-clinical voice hearers were compared in terms of the nature and frequency of their experienced trauma (Andrew et al., 2008). Clinical voice hearers reported significantly more incidences of childhood sexual abuse than non-clinical. Seventy-eight percent of clinical voice hearers also demonstrated symptoms indicative of DSM-IV post-traumatic stress disorder (PTSD). This suggests that perhaps severity of abuse and the emotional response may be distinguishing factors predicting clinical status. Subsequent research has suggested a dose-dependent relationship, with the greater the amount of trauma (severity and/or new events) associated with an escalation in risk of voice hearing (Bentall et al., 2012). For example, Whitfield et al. (2005) reported a 5-fold increased risk for AVH in adulthood for those who have experienced over seven adverse childhood experiences, compared to those who have experienced none at all. Similarly, Shevlin et al. (2011) reported that respondents who had experienced three different types of trauma (sexual and physical assault, rape) were eleven-times more likely to develop AVH compared to their trauma-free counterparts.

The experience of trauma as a vulnerability factor leading to hallucinatory experiences has been illustrated from child populations through to adulthood. Combining several risk factors from enduring vulnerabilities, proximal life stressors and dysfunctional psychological coping strategies, Goldstone et al. (2012) showed that childhood emotional trauma, metacognitions and life hassles all predicted the presence of auditory hallucinations in a non-clinical sample. Past research has found sexual trauma to be explicitly predictive of hallucinatory experiences (Kilcommons and Morrison, 2005; Kilcommons et al., 2008), yet this model was only able to account for 20% of the variance in vulnerability to AVH specifically. Being one of the strongest predictors of AVH, emotional trauma cannot be discounted. However, the experience of trauma *per se* has also been shown to increase sensitivity of an individual to other life stressors (Read et al., 2001), which mirrors the previously mentioned findings of increased trauma leading to greater susceptibility to AVH (Whitfield et al., 2005; Shevlin et al., 2011). This increased sensitivity in and of itself could enhance the likelihood of clinical symptom development in an already at risk population, with the interaction of traumatic effects predisposing individuals to a heightened risk of AVH development.

One way in which trauma may act on the individual to result in AVH experiences is via intrusive thoughts and the re-experiencing of traumatic memories. The association between AVH and PTSD is well documented (e.g., Braakman et al., 2009; Anketell et al., 2010; Soosay et al., 2012). Characteristic of DSM-IV PTSD, these AVH experiences have many phenomenological similarities with “flashback” symptoms: uncontrolled revisiting of the traumatic experience (Morrison et al., 2003). A specific diagnosis of PTSD has been argued to depend on the individual's level of awareness of the intrusive thoughts and its link to previous trauma (Steel et al., 2005). There have been instances in which the intrusive memories relate directly back to the trauma (Hardy et al., 2005). However this only occurred in a minority of cases analysed, which suggests that the relationship between trauma and AVH is not usually so clear-cut. The role of dissociation in mediating this relationship has been extensively investigated (e.g., Perona-Garcelán et al., 2012; Varese et al., 2012) and may help to account for the “separateness” or “otherness” which leads to experiences being subjectively appraised as hallucinations and not thoughts or memories (Perona-Garcelán et al., 2011; Longden et al., 2012). Other factors, such as high levels of unusual and schizotypal beliefs have also been shown to impact on the experience of intrusive thoughts resulting from trauma (Berenbaum, 1999).

Another factor identified as impacting on the existence of AVH after trauma is negative schematic beliefs that exist about the individual and others around them (Gracie et al., 2007). Negative schemas come about via the social and emotional learning of an individual (Birchwood, 2003). In voice hearers who have experienced trauma from an early age, these schemas can become ingrained over a long period of time. As these beliefs feed into and encourage the development of delusions (Freeman et al., 2002) it seems likely that they may also predispose certain people to develop AVH. Although this is one avenue which still requires more thorough research, the relation of negative schemas to the re-experiencing trauma-related AVH seems to be one facet which may be preventable through a targeted intervention.

The examination of childhood trauma predisposing AVH is often assessed through retrospective recall. This invites the possibility of factors that may impinge on the reliability of traumatic memories. Bias in recall can occur through: repression (Colangelo, 2009), suggestibility of the individual reinforced through practices such as leading questions and hypnosis (Andrews et al., 1999), the need to rationalize the presence of AVH (Schacter, 2001); and, for clinical patients: delusions (Young et al., 2001) and cognitive deteriorations (Driesen et al., 2008). Although these should be kept in mind when examining the veracity of self-reported trauma and AVH, it should also be noted that research indicates a strong tendency to under- instead of over-report abuse in psychiatric patients (Spataro et al., 2004; Fisher et al., 2011). The reliability of retrospective reports of childhood abuse has been tested, with high levels of concurrent validity and test-retest reliability for adult retrospective abuse accounts compared with clinical case notes (Fisher et al., 2011). Similar findings have been gained for female clinical patients (Meyer et al., 1996) and the retrospective accounts of child abuse in patients diagnosed with schizophrenia or bipolar disorder (Goodman et al., 1999). Additionally, the prospective epidemiological research tends to support those data collected through subjective rating scales. Corroborating documentation from independent parties can also be used to increase the confidence in data collected.

## Significance of the schizotypal personality trait

### (Child, childhood, adolescent, adolescence, early adulthood, adult, auditory hallucinations, auditory perceptions, voice hearing, psychotic experiences, psychosis, clinical, non-clinical, schizotypy, schizotypal, continuum)

Under a continuum model of psychosis, schizotypy is believed to represent a trait-like marker of schizophrenia personality which is evident in the general population (Johns et al., 2004). Although the continuum model is not regarded in the current review as a dominant framework of causation for AVH, schizotypy is readily regarded as a biological precursor for hallucinatory experiences, with a common etiologic component being identified between hallucinatory symptoms and schizotypy in non-clinical (Mata et al., 2000, 2003) and clinical (Grove et al., 1991; Kwapil, 1998; Gooding et al., 2005) groups. Accordingly, an increase in this personality trait has been conceptualized as part of the at-risk mental health criteria (ARMS; e.g., Wood et al., 2011). Individuals who score highly on schizotypy are more likely to display a propensity for anomalous experiences including AVH (e.g., Barkus et al., 2007). It involves qualities such as odd behavior, unusual perceptual experiences, aloofness, introversion, and cognitive disorganization (Raine, 2006). The personality trait is reported to decrease with age (Rössler et al., 2007), being at its peak in adolescence (Fossati et al., 2007), although there are limited investigations of its base rate in children. The most robust difference of healthy voice hearers compared to the general population is a significantly increased level of overall schizotypy (Sommer et al., 2010). Since AVH are a positive symptom of psychotic illness voice hearers would be expected to display a significant increase in positive schizotypy only, as it is a trait vulnerability for the experience of hallucinatory phenomena (Tsakanikos and Reed, 2005). However, the difference between healthy voice hearers and controls reflects a general increase in all schizotypal dimensions. This could be indicative of the presence of AVH being associated with subclinical levels of all schizotypal phenomena. In combination with an increased family loading for psychosis (Sommer et al., 2010), these findings may be suggestive of a genetic predisposition for psychosis for those experiencing AVH who have increased schizotypal levels and a genetic labiality. Evidence for an etiologic component linking hallucinatory predisposition and schizotypy has also been illustrated by Mata et al. (2003) through the identification of relatives of psychotic patients who display significantly elevated schizotypy levels compared to controls. In adolescents, positive schizotypy and anxiety have been reported to have a reciprocal relationship, both of which increase the likelihood maladaptive metacognitions will be present (Debbané et al., 2012). With the exception of controllability of thought, many of the maladaptive metacognitions are common to both ARMS patients and high schizotypes who hallucinate (Barkus et al., 2010).

Healthy individuals high on the schizotypal dimension have also been found to share a degree of liability toward AVH under experimental conditions. In these studies, under ambiguous conditions, healthy individuals high on the schizotypal personality dimension are shown to be significantly more likely to report the existence of some sort of auditory perceptual experience in the absence of any such corresponding stimuli (Tsakanikos and Reed, 2005; Barkus et al., 2007, 2010; Galdos et al., 2011). Although these individuals do not actually experience AVH, their pattern of responding is consistent with that of those healthy individuals who do experience AVH (Haddock et al., 1995). It seems then, that some shared cognitive component (for example, the externality hypothesis; Garety et al., 2001) may be responsible for the biased attributional process present in both schizotypal and AVH populations.

## Neuroimaging studies

### (Early adulthood, adult, auditory hallucinations, auditory perceptions, voice hearing, psychotic experiences, psychosis, clinical, non-clinical, neuroimaging, fMRI)

Recently, studies examining the distribution of brain regions recruited during AVH have flourished. The development of functional imaging techniques have allowed the capture of the brain activation related to AVH. As these techniques become more refined, we are able to pinpoint activation patterns as the hallucinatory symptom is being experienced. This allows activation patterns to be documented and studied in order to gain a better understanding of the biological mechanisms underpinning AVH. By studying these biological mechanisms we are able to gain a more precise understanding of the neurological changes that occur leading up to, during and in the cessation of hallucinatory phenomena. In a recent meta-analysis Jardri et al. (2011) noted several brain networks to be activated (fMRI, PET) during AVH, including fronto-temporal brain regions, and hippocampal/parrahippocampal regions. Allen et al. (2008) also noted the involvement of the prefrontal premotor cingulate, secondary auditory cortex, Heschl's gyrus (primary sensory cortex), anterior cingulate, middle and superior temporal gyri, cerebellar areas, and aberrant activation from emotional attention centers such as the rostral/ventral anterior cingulate. In another meta-analysis, Kompus et al. (2011) compared neuroimaging (fMRI, PET) findings for patients with schizophrenia while they process external auditory stimuli, to studies of patients experiencing AVH in the absence of any external auditory stimuli. A paradoxical brain activation in relation to AVH was noted, such that there exists an overlap in the activation of the left primary auditory cortex and right rostral prefrontal cortex. These areas display increased activation in the absence of external stimuli (AVH) and decreased activation when an external stimulus is actually present. The authors deduced that an attentional bias may exist in patients who experience AVH so much so that attention is focused predominantly on internally generated information. This is significant in that the mechanism underpinning AVH occur could be explained by a bias in the cognitive processing of auditory stimuli. As this mechanism deserves a level of detail which is beyond the scope of this paper, a comprehensive review of the area has been conducted by Badcock and Hugdahl (2012).

There has been less of an emphasis on the investigation of non-clinical AVH using imaging techniques. Barkus et al. (2007) revealed that non-clinical experimentally elicited hallucinations broadly activate the same regions associated with AVH in patients with schizophrenia. This study requires replication in a larger sample size. Diederen et al. (2012) is the only neuroimaging study to date which compares AVH across clinical and non-clinical groups. Using fMRI, several areas were found to be significantly activated for both groups while experiencing AVH, including: superior temporal gyri, insula, bilateral inferior frontal gyri, inferior parietal lobule, left precentral gyrus, right cerebellum, and superior temporal pole. Significantly, no differences were found between non-clinical and clinical groups, suggesting the same brain regions are involved for all AVH. Also, the brain regions activated during AVH are the same as those which have been documented for AVH in schizophrenia and other previous research [for review see Allen et al. (2008)].

The focus in many imaging studies to-date has been on AVH in patients with schizophrenia or the investigation of non-clinical correlates such as imagining speech. One study has investigated the differences between patients with Parkinson's disease who do and do not experience AVH (Matsui et al., 2006). In contrast to many of the fMRI studies in patients with schizophrenia they reported hypoperfusion of the bilateral prefrontal cortex and right superior temporal gyrus in those with AVH. These are similar areas to those seen in patients with schizophrenia who experience AVH, however the mechanism appears to be about reduced rather than increased activation.

Given that AVH occur in other disorders besides psychosis consideration of these needs to be included in a comprehensive review. Some reference has been made to other disorders in the previous text; below we will give consideration of disorders which could be seen as providing an altered state of consciousness leading to ripe conditions for AVH to occur.

## Epilepsy

### (Adult, auditory hallucinations, auditory perceptions, voice hearing, psychotic experiences, psychosis, clinical, non-clinical, epilepsy, neurological abnormality)

Apart from AVH in healthy and psychological clinical groups, AVH have also been reported to occur in temporal lobe epilepsy (Brasic and Perry, 1997; Hug et al., 2011; Hauf et al., 2012). As a neurological disorder, epilepsy can create the biological threshold under which hallucinatory symptoms develop due to neurological abnormalities [such as hyperfusion of the primary auditory cortex (Hauf et al., 2012)]. The manifestation of AVH in epilepsy form part of a unique trajectory that is believed to be separate again from that of psychotic AVH or non-clinical AVH. Evidence supporting this model is seen in studies comparing AVH in psychosis to those experienced in temporal lobe epilepsy. Through such a comparison, it can be seen that auditory phenomena are usually perceptually lateralised to the left side in epilepsy patients, with this relationship not evident in psychotic groups (Clarke et al., 2003; Florindo et al., 2006; Hug et al., 2011). As such, a clinical differentiation between AVH experiences in epilepsy and psychotic groups seems likely. Prevalence rates of hallucinatory experiences range from 3.3% in epilepsy generally, to 14% in temporal lobe epilepsy specifically (Torta and Keller, 1999). Phenomenologically, these occurrences mirror those documented in clinical (schizophrenia/psychosis) groups, with hallucinations ranging in complexity from ringing and tonal sensations, right through to more complex phenomena including musical and melody perceptions and AVH of human voices (Hug et al., 2011). Patients with epilepsy also demonstrate similar results on behavioral and neuroimaging analyses when compared to patients with schizophrenia, providing further evidence for AVH existing trans-diagnostically, that is, a symptom independent of diagnostic categorization. That the neuropsychological and neuroimaging indices of AVH in epilepsy are similar to those found in schizophrenia (Korsnes et al., 2010) lends weight once more to AVH being orthogonal of any one diagnosis.

One factor that is specific to temporal lobe disturbances is the high frequency of religious and mystical experiences reported (Ozkara et al., 2004). In the context of AVH experienced during temporal lobe epilepsy, this means that patients reporting such experiences can often attribute them to some sort of religious sensation or clarity of experience relating to God or spirits (Åsheim Hansen and Brodtkorb, 2003). This may include hearing a voice telling the individual to kneel and worship God (Ogata and Miyakawa, 1998), hearing hallucinations of God's voice (Åsheim Hansen and Brodtkorb, 2003), hearing the repetition of a religious expression (Ozkara et al., 2004) or the AVH may be regarded as a prophecy or deeper message being conveyed to the individual (Åsheim Hansen and Brodtkorb, 2003). The experience of these religious AVH for individuals with temporal lobe epilepsy does not usually result in distress or discomfort. Rather, they appear to mimic AVH in healthy voice hearers, so much so that any dysfunction that may occur is usually associated with other phenomena taking place in that individuals life (for instance the epileptic seizures or reduced quality of life associated with severe temporal lobe epilepsy). What differentiates these AVH from those experienced in healthy voice hearers is the intense religious experience which occurs. This seems to be inextricably linked to the right temporal lobe seizures which take place during and after these spiritual events (Devinsky and Lai, 2008).

## Substance-induced auditory verbal hallucinations

### (Adult, auditory hallucinations, auditory perceptions, voice hearing, psychotic experiences, psychosis, clinical, non-clinical, substance induced, cannabis, cocaine, amphetamines, opiates, illicit drug use, drugs)

AVH are also frequently documented under substance-induced states. Under the biopsychosocial model of AVH development, whilst illicit substance use is conceptualized as being an environmental and psychologically motivated facet (via substance abuse and addiction models; Cavaiola, 2009), the causes are on a biological level. The ingestion of illicit substances into the body acts by altering neurotransmission and, with persistent and pernicious use, may lead to structural and functional changes in the brain. The subjective effects of substances such as cannabis are subject to individual variability which may be accounted for by biologically meaningful phenotypes such as schizotypy (e.g., Stirling et al., 2008). Even considering the well-documented relationship between cannabis and psychosis, cannabis is considered a component cause which operates against a background of other risk factors (Castle, 2013). Hallucinations experienced in these states can be acute and transitory, passing once the substance ceases activation in the body, or they can be more chronic and ingrained-possibly leading to the development of later psychoses (Barkus and Murray, 2010). It is not known however what features differentiate individuals who do experience AVH in these states compared to those that do not. Several studies concerned with different classes of illicit drug have found that use of these substances, specifically: amphetamines (e.g., Ujike and Sato, 2004; Akiyama, 2006), cannabis (e.g., Arseneault et al., 2002; Semple et al., 2005) and cocaine (e.g., Karila et al., 2010) predate the onset of psychotic symptomatology, including AVH. The experience of psychotic symptoms (delusions and auditory hallucinations) has been reported in over half of a sample of cannabis and cocaine substance abusers, either during the use or withdrawal of those substances (Smith et al., 2009). Dependent users of cocaine (Mahoney et al., 2008), opiates (Smith et al., 2009), methamphetamine (Srisurapanont et al., 2003; Auten et al., 2012) and cannabis (Arendt et al., 2005) also experience delusions and AVH (Smith et al., 2009), usually in over 50% of responses for each study. The severity of the psychotic symptoms are almost always significantly related to the rate of substance use, such that severity of psychotic symptomatology increased with increasing rate of dependence. This association mimics a dose-dependent relationship (Thirthalli and Benegal, 2006) between the rate of substance use and the severity of psychotic symptom presentation.

In those using cocaine specifically, the AVH experienced are usually always quite vivid, isolated, and associated with the thought content at the time (Roncero et al., 2012). More often than not this is a state of paranoia, such that the person believes they hear footsteps and are being followed by spies, as an example. AVH have also been documented in cocaine users after the high has subsided (i.e., during abstinence), with this condition significantly more likely to occur in women compared to men (Mahoney et al., 2010). The reason for this difference between the sexes is still yet to be investigated. Interestingly, not all studies report an association between illicit substance use and AVH, with Ohayon (2000) reporting a significant association with the use of drugs (opiates, cocaine, amphetamines) and all hallucinatory phenomena (visual, tactile, gustatory, haptic) except auditory. Given the magnitude of this population based study (12,500 + participants), such results are surprising and warrant further investigation. It may be that other variables related to schizophrenia, such as schizotypy scores, are involved in increasing the likelihood that AVH will occur after illicit substances (e.g., Barkus et al., 2006; Barkus and Lewis, 2008).

## Hypnagogic and hypnopompic experiences

### (Adult, auditory hallucinations, auditory perceptions, sleep related, perceptual experiences, sleep, hypnagogic, hypnopompic, phenomenology, sleep experiences)

One of the most common instances in which healthy individuals experience AVH is during the reduced level of consciousness associated with falling asleep (hypnagogic) and waking up (hypnopompic) (henceforth H/H experiences). Both these experiences are believed to represent the same group of phenomena (Mavromatis, 1988). In a relatively recent review, Ohayon (2000) found that 25% of people from the general population reported having a hypnagogic experience, whilst 18% reported a hypnopompic experience. Although significantly more common in the general population than conscious AVH, the fact that these experiences only occur in certain individuals lends to the idea that some biological factor may be mediating this relationship. Recent research has identified significant associations between dissociative personality traits and H/H experiences, which may be able to account for the predisposition to such experiences (Koffel and Watson, 2009). Disruptions to the sleep-wake cycle have been found to intensify dissociative symptoms (Giesbrecht et al., 2007), and interestingly, increased levels of dissociation and schizotypy have been found to be common in those with H/H experiences (Watson, 2001; Koffel and Watson, 2009). In student populations prevalence rates of H/H experiences have been documented to be as high as 85% (Jones et al., 2009). Similarly, rates of dissociative tendencies have also been found to decline with age in adulthood (Torem et al., 1992), which provides further support for the biological link between dissociative personality traits and H/H experiences. These experiences usually include any one of the following phenomenological characteristics; a person's name being called, reference to a past conversation, meaningless words, quotes and remarks directed at the individual (Mavromatis, 1988). When compared to the AVH experienced during wakefulness, there are some common features present. Just like AVH, they are more likely to be the voice of someone known to the person, speak directly to the person, and are more likely to be affect neutral (like those of healthy voice hearers) (Jones et al., 2010). Different to those of AVH in wakefulness however, are findings of unclear voices, contrary to the usual clearly audible voices heard in AVH, and unlike voice hearing in the general population, command AVH are only reported around 4% of the time (Jones et al., 2010). The link between H/H experiences and AVH in wakefulness, however, is still unclear. Recent research has uncovered intrusive auditory imagery as a significant predictor in the onset of H/H experiences (McCarthy-Jones et al., 2011). This is reflective of cognitive models of clinical AVH, whereby intrusive thoughts and source monitoring errors have been implicated in the onset of hallucinatory experiences (Morrison and Baker, 2000). Could it be that the two share underlying biological mechanisms such as a lack of cognitive inhibitory control, or a possible loss of the agency of self vs. other in relation to cognitions? Likewise, it is not known whether H/H experiences could represent a precursor or vulnerability marker for the subsequent development of more pathological hallucinatory experiences. As this area is still in such early stages of understanding, further research aimed at elucidating the significance of H/H experiences is warranted.

## Hallucinatory experiences in the elderly

### (Adult, late adulthood, elderly, senior, auditory hallucinations, auditory perceptions, psychotic experiences, voice hearing, psychosis, clinical, non-clinical, music hallucinations, alzheimer's, dementia, neural degeneration, bereavement)

Interestingly, prevalence rates of hallucinatory experiences have been shown to increase when adults reach an age of 60 and over (Tien, 1991; Turvey et al., 2001), with factors such as the loss of a spouse, neurocognitive degeneration and sensory deficits being implicated as risk factors for the elderly (Grimby, 1993, 1998). The types of hallucinations found to occur in the elderly are often slightly different to those in the general population, with visual hallucinations more frequently reported, as well as musical hallucinations, where the hearer experiences tunes or harmonies instead of voices (Berrios, 1990). The development of brain disease, hearing loss and advanced age are shown to be significantly related to the experience of musical hallucinations (Stephane and Hsu, 1996; Tanriverdi et al., 2001; Cole et al., 2002).

Whilst visual hallucinations are more commonly reported in dementia there are also studies which speak to the prevalence of auditory hallucinations. Auditory hallucinations occur at a rate of 6.8% in elderly populations diagnosed with Parkinson's disease; with depression and sleep difficulties being predictive of those psychotic symptoms, over and above motor or cognitive symptoms (Lee and Weintraub, 2012; Mack et al., 2012). The experiencing of auditory hallucinations in Parkinson's disease is believed to be related to the pathophysiology of the disease, not merely a side effect of medication (Fénelon, 2008). Auditory hallucinations are also found in Alzheimer's and Lewy body dementia (Ballard et al., 2001). In Alzheimer's the presence of hallucinations may be indicative of a more rapid progression of symptoms (Förstl et al., 1993). Within an institutional setting the reports of hallucinations may be related to environmental factors such as lack of entertainment or perceptual stimulation (e.g., Cohen-Mansfield and Golander, 2012). That AVH are present in dementias, where marked neural degeneration is occurring, points to the neurologically driven nature of these experiences. It seems that environmental factors or the co-occurrence of other psychiatric symptoms increases the likelihood they will occur, suggesting other symptoms share common underpinnings or that one may exacerbate the other.

Although there is still a major deficit in knowledge concerning the experience of hallucinations in the elderly, it has been shown that musical hallucinations can progress into AVH over time (Cole et al., 2002; Fischer et al., 2004). Such a transition is indicative of a decline in psychological functioning, and is found to be associated with a decline in treatment response as well as degree of insight of the affected individual (Fischer et al., 2004). It seems then, that the hallucinatory experiences in the elderly may be more attributable to sensory loss and decline in cognitive functions, rather than those factors and traits discussed previous which predispose those of a younger age (see Tanriverdi et al., 2001; Evers and Ellger, 2004; Sanchez et al., 2011). Thus, the aetiology of AVH in the elderly compared to the young may differ; further evidence for this is demonstrated in the effective treatments of elderly patients which are anti-convulsant and anti-depressive rather than antipsychotic in nature (Evers and Ellger, 2004; Larøi et al., 2005; Cope and Baguley, 2009).

For those elderly individuals who have experienced the loss of a spouse however, AVH are much more common, occurring in up to one third of the bereaved (Grimby, 1993). The hallucinations occur in the clear conscious state, and are often reported as being comforting and positive to the individual, rarely causing distress. As these hallucinatory experiences occur during times of excessive emotion, confusion can come about, often resulting in reports of the person believing their spouse is actually present (Grimby, 1993). Hallucinations occurring out of bereavement have not been found to ascribe to any definitions of pseudo-hallucinations (Baethge, 2002). Similarly, they do not meet any diagnostic criteria for psychological disorders. As a result, the existence of this type of hallucinatory experience seems to be in line with those of healthy voice-hearers, in that they do not cause any sort of distress, and thus, do not warrant any sort of clinical intervention (Pierre, 2010).

## Methodological caveats

### (Child, childhood, adolescent, adolescence, early adulthood, auditory hallucinations, auditory perceptions, voice hearing, psychotic experiences, psychosis, clinical, non-clinical, hep seeking)

As with many areas within psychiatry, the studies investigating auditory hallucinations in both psychiatric and healthy volunteers have their limitations. Some are attributable to the subjective nature of the experiences, whilst others can be considered directly related to the methodologies used.

In relation to studies assessing outcomes for those voice hearers who experienced childhood trauma and significant distress, there exist a number of issues limiting research findings. First, the reliance on cross-sectional methodology, although providing a wealth of current information, fails to provide reliable information on antecedents to psychological dysfunction without the probable interference from retrospective bias. Furthermore, this methodology limits the conclusions that can be drawn relating to precursors of voice hearing, prevalence of AVH at different time points and the differential patterns of clinical outcome for voice hearers. Studies in children who have been abused are often from a retrospective standpoint which ensures they are focused on one illness outcome such as psychosis. Given the multiple exposures to adverse psychopathology associated with abusive childhood environments (ambiguous communications, substance abuse, parental psychopathology and neglect to name but a few) there are many factors which need to be taken into account, and are often ignored, in the current literature. Retrospective studies, as discussed early on, come up against similar pitfalls, specifically concerning retrospective recall and bias in recall, especially when memories are sought over a decade later.

Another issue that needs to be considered across studies is the specificity of what is considered an auditory hallucination. Psychiatrically, an auditory hallucination is a sensory experience which: 1. occurs in the absence of any external stimulation; 2. takes place with sufficient conviction such that it is considered reality; and 3. occurs outside of conscious control (David, 2004). Yet it is unclear then, whether auditory events such as hearing tones, or “noises” (Shevlin et al., 2007) other than voices should be classified as AVH. It is possible that different definitions of AVH may bias results, and prevent replication across studies. Inclusion of more lax criteria under the perception of an auditory hallucination may result in an inflated prevalence of voice hearing for some studies and could limit the reliability of those results, particularly when generalizing from healthy voice hearers to clinical voice hearers. Given that we use many of the findings from healthy voice hearers to understand the mechanisms occurring in clinical samples, this raises concerns about the relevance and validity of some of the cognitive models proposed. Perhaps the time has come to form a consensus on what is understood to comprise an auditory verbal hallucination so as to minimize any further discrepancies across studies. This may include determining under what circumstances non-clinical AVH provide useful information for clinical samples both in terms of underlying mechanisms and protective factors from help seeking behaviors.

Another factor particularly pertinent in comparing clinical voice hearers to those who do not have a psychiatric diagnosis is the separation of voice hearers into their respective research groups. For instance, it is unknown whether “healthy” voice hearers are in fact just that, or whether they are reflective of the prodrome period before psychosis onset. Similarly, categorizing people as healthy voice hearers simply because they do not experience any distress or have any other psychotic symptomatology associated with their AVH is inherently simplistic; especially considering the large variations in frequency, duration and number of voices each individual presents with. Is it so that someone who hears voices once a week can be understood to be categorically the same as someone who hears voices four times a day, simply because there is no other associated distress or dysfunction? Further research aimed at understanding the implications of these phenomenological differences is required before categorization of voice hearers as simply “clinical” or “healthy” is unduly accepted. This limitation is partly driven by the diagnostic categories emphasis on help seeking and reduced capacity to function in day-to-day life as defining whether someone meets the criteria for being a patient. The requirement of functioning in a day-to-day manner is highly subjective in and of itself. For example; many of the components of schizotypal personality disorder are likely to coincide with “healthy voice hearing,” yet one is classified as a disorder whilst the other is not. Perhaps the conceptualization of diagnostic manuals with a greater emphasis on the continuum approach will assist this to some degree. However, it is recognized that the prime mechanism for people receiving treatment will always be whether they are help seeking themselves or put in a position where they are compelled to seek help.

Finally, as this review has delved into the multitude of AVH presentations across the lifespan and in different modalities of dysfunction, we want to drive home the importance of considering these hallucinatory experiences as a symptom in isolation of any specific psychiatric diagnosis. As is evident by the research presented, AVH are very similar in many respects in clinical and non-clinical groups, however they also differ on considerable key areas. The importance of this phenomenological diversity cannot be overlooked, and should be understood in terms of a symptom that is heterogeneous and may depend upon the population it occurs in. The implications for this in terms of conceptualizing a model of AVH comes back to a question of whether AVH should be recognized as an independent symptom in and of itself, or whether it should be classified as part of specific disorders diagnostic criteria. Although research findings delving into phenomenological comparisons of AVH across psychiatric and non-clinical populations are valuable, further research concerned with the changing dynamics of AVH presentation, as well as its association with other symptoms such as delusions and dissociation (outside of just schizophrenia research) is necessary.

## Clinical implications

Before drawing any firm conclusions about the mechanisms which underlie AVH, and how these mechanisms can be targeted in future clinical research it is important to consider that AVH take many forms. Some (and often the most severe) are associated with psychotic illnesses, others are understood to be manifestations of neurological disorders or substance induced, whilst others again are conceptualized as a type of healthy coping mechanism which comes about as a result of traumatic and distressing event(s). The very distinct nature of each of these manifestations of AVH implies that we should not consider them as a single category, and the heterogeneity of AVH should not be ignored. It appears that it is this heterogeneity in the presentation of AVH across different groups of the population which may make them diagnostic specific. What we see emerging as a common pattern in clinical groups is that distress is a defining factor determining need for care. The presence of voice hearing in and of itself is not sufficient for deteriorating functioning. As is evident in the review of non-clinical research, AVH usually do not cause distress or impair functioning in these voice hearers. Thus, is it not the experience of voice hearing in and of itself that leads to a decline in functioning, but the associated distress. The implications of this are two fold. First, we need to stop referring to AVH and psychotic disorders as interchangeable features. The fact that AVH occur in a multitude of different psychological, neurological and substance-induced disorders, as well as the general healthy population, means that AVH should no longer be recognized as a diagnostic indicator. Secondly, we need to start conceptualizing AVH as a symptom unto itself, and not intrinsically related to the outcome of the patient or individual. As AVH should not be understood as a diagnostic marker, it then follows that AVH have no bearing on the functional outcome of the individual. The presence of AVH in an individual by no means indicates poor functioning, but the level of functioning is one of the most pivotal markers of whether their experience of AVH is associated with pathology. Functioning is connected intricately with the level of distress experienced by the voice hearer but also is reflected in the emotional regulation of the individual. As has been suggested previously, this has an important impact on how different cognitive mechanisms interact in the voice hearing experience, to either function as a healthy coping mechanism for the individual, or create dissonance and associated distress, requiring clinical intervention.

Moving forward from these implications toward suggestions for the clinical utility of these findings first requires a focus on distress as a central component in dysfunction associated with AVH. As such, cognitive-behavioral therapy centered around reducing the distress caused by voice hearing should become a priority, over and above current models which instead place emphasis on the perceptual phenomena of voice hearing (Fowler et al., 1995; Thomas et al., 2011). The persistence of AVH was also found to be associated with the cognitive and emotional interpretations of the voice, specifically through the formation of delusions, and associated anxiety, paranoia and depression. Targeting and challenging negative schemas which exist in combination with AVH may aid in reducing the associated distress elicited by AVH in clinical groups. Finally, cognitive-behavioral therapy whereby both the client and therapist collectively dispute irrational delusions associated with AVH may also help to alleviate distress and associated dysfunctional cognitive components.

However, it needs to be acknowledged that distress is not the only component which determines a need for care in people who experience AVH. For instance, PTSD is a psychological disorder associated with significant rates of distress when compared to associated pathologies. Yet AVH are only experienced by a subgroup of patients (Anketell et al., 2010). Therefore, distress cannot be the sole predictor of clinical dysfunction. It may be that the period of AVH onset is a defining feature of whether or not that experience becomes pathological. For instance, we have seen that those AVH which follow onwards from trauma may be more likely to be followed by some sort of psychosis. Contrastingly, those voice hearing experiences which occur in childhood quiet often abate once the child enters adolescence, suggesting that the experience of voice hearing is not an indicator of problematic development. It may be that AVH are used by the human brain as some sort of coping mechanism in response to change, distress, or stress. That is, it is not the perceptual experience of voice hearing *per se* that leads to pathology-it is the associated features, or other dysfunctional mechanisms in the individual (the factors labeled background features in our framework provided in Figure 1). If the individual is not able to regulate and respond to their challenges and stress in an emotionally cohesive way this creates dissonance, which may result in the progression to pathology. Such a conceptualization is purely theoretical, and further clinical research is necessary to disentangle our understanding of clinical voice hearing from that which occurs in the healthy, functioning individual. Longitudinal research on a large population scale would be optimal to unravel the connection which exists between stressful life events and subsequent voice hearing. Additionally, research focused on the coping mechanisms of these individuals, whether it be cognitive, behavioral or emotional, would also benefit our understanding of the clinical consequence of voice hearing perception in the manifestation and maintenance of AVH.

Finally, it seems clear that an understanding of the phenomenology of clinical voice hearing as a symptomatic component of psychosis has reached a stage of competent understanding. Perhaps the time has come for psychosis research to begin focusing on stable risk components such as schizotypy, rather than symptoms like AVH. It has become clear that AVH are a transdiagnostic symptom which cannot give us an indication of outcome, especially one specific to psychosis. In clinical staging models (Wood et al., 2011) early phases must focus on stable rather than transitory features of pathology which are able to separate high risk individuals from their counterparts. Clinical features such as AVH seem no longer able to provide us with such a distinction. As a result, a move toward early indicators of risk, such as neurological soft signs and schizotypy appear to be a much more feasible line of enquiry.

## Conclusions

The phenomenology of AVH reveals individual differences in the experiences which are unsurprising given their subjective nature. The clarity and authenticity of AVH ensures that they have some impact on the lives of those who experience them; a differential factor is whether this influence is positive or negative. Even when considering short lived transient phenomena such as hypnogogic and hypnopompic experiences, the often frightening and alarming nature of the experiences can reduce affect in the proceeding hours. The clinical relevance of AVH under conditions of reduced consciousness needs further investigation. From considering the factors which precipitate AVH in childhood, adolescence and adulthood two points become evident. First, there is a consistency in environmental and social factors thought to trigger AVH across all ages. This suggests that these phenomena are comparable across the lifespan which has diagnostic and therapeutic implications in itself. Additionally, it may point to the underlying biological and cognitive mechanisms underpinning AVH being consistent regardless of age. Second, there is a paucity of research considering adolescence as a separate vulnerability group. Despite the field acknowledging the rapid social, psychological and neurobiological changes associated with adolescence, there are few papers considering them a group of unique interest. These studies are of particular importance given the need to identify factors which lead to the persistence of AVH from childhood into adulthood and elucidating which factors cluster to differentiate clinically at risk samples from those who will remain psychologically intact. These questions require investigation and addressing to facilitate prevention and early intervention in those at risk for serious mental health disorders.

Returning to Figure 1 to reconsider the biopsychosocial framework used to outline this literature review. Many of the AVH attributable to biological causes such as epilepsy or dementia are treated as symptoms to be managed and/or eliminated from what is a complex neurological picture. The content of voices is often ignored and not engaged in a therapeutic manner; given the biological nature of the cause, interventions are frequently pharmacological in nature. Additionally, substance induced and sleep related phenomena are equally dismissed given the altered states involved. Individual differences in the experiences reported after using substances such as cannabis may in fact be informative. Given the pharmacologically “dirty” nature of many drugs of abuse, any pre-existing dysregulation in neurobiology will be further exacerbated by substance use and could account for the variation in experiences reported. Similarly sleep disturbances may be indicative of other vulnerabilities particularly when the associations between sleep problems and psychiatric conditions are kept in mind.

The cognitive mechanism and factors associated with AVH are clustered to provide attempts to explicate the processes underpinning AVH which may be ameliorated by psychological therapies such as cognitive behavioral therapy. The difference between clinical and non-clinical groups lies in the heterogeneous way in which each respective group processes their experiences. Consequently the identification of common mechanisms underpinning both clinical and non-clinical AVH may highlight protective factors. The most notable psychological factor which differentiates clinical from non-clinical experiences of AVH is affect regulation and subsequent negative interactions with the voices heard. Degree of preoccupation and distress associated with voice hearing is consistently associated with help seeking behaviors regardless of age or origin of the AVH. Interestingly, distress attributable to AVH is still found in those underpinned by neurological disorders despite the origins being concrete in nature. This suggests that the distress and preoccupation may be attributable to other accompanying factors and may interact with the cognitive mechanisms underpinning AVH. An alternative explanation may be that the experience of voice hearing is the end point of a number of causal pathways. Given its lack of specificity as a diagnostic symptom this may be the case. Perhaps the focus of future research should therefore be in trying to reduce the heterogeneity of the experiences by more closely defining the phenomena under consideration. Once experiences are more closely defined the multiple and complex etiological factors of AVH could be subclassed according to type of experience. For instance the causes of persisting imaginary friends in adolescents could be different to the first presentation of a voice in early adulthood. Do AVH cognitive mechanisms in individuals exposed to trauma differ from those without traumatic experiences who have high schizotypy scores? The competing cognitive explanations of AVH may be focusing in on different sub-classes of experiences. This requires further refinement and investigation.

It is likely that social and environmental factors interact with more stable vulnerability factors to lead to need for care in those who experience AVH. The key point with these factors is that they are malleable. They lack the hidden unknown qualities associated with genetic, biological and cognitive risks for AVH. As a simple example, providing sensory stimulation to elderly residential care settings would reduce the likelihood of AVH occurring. Developing targeted interventions to improve coping and stress responses to life events and even trauma may help vulnerable individuals interact with their voices in a less distressing manner. Interventions targeting stress and affect regulation can often be delivered in a group setting without the stigmatization associated with “therapy” in the formal sense.

With the aim to improve the outcomes for young people who are at risk for developing serious mental health disorders further research is needed to determine the factors which predict persistence and need for care. Identifying the cluster of factors which produce differential developmental trajectories from childhood through to adulthood will be essential to assist earlier identification of those at risk. A move away from the consideration of triggers for AVH in isolation needs to happen for this to be achieved. Additionally research needs to become focused on the mechanisms underpinning relationships between variables. For example, the relationship between AVH and trauma is well documented; research now needs to move to determine why this relationship exists and what other factors co-occur which may be relevant to determining need for care. Recognition that AVH are not exclusive to schizophrenia-spectrum disorders needs to pervade the literature. Researchers interested in this as an end point need to now begin to refine their phenotyping to ensure that they are examining a non-clinical “psychosis-enriched” healthy volunteer sample or relevant clinical samples.

### Conflict of interest statement

The authors declare that the research was conducted in the absence of any commercial or financial relationships that could be construed as a potential conflict of interest.
